# How many familial relationship testing results could be wrong?

**DOI:** 10.1371/journal.pgen.1008929

**Published:** 2020-08-13

**Authors:** Jianye Ge, Bruce Budowle

**Affiliations:** Center for Human Identification, University of North Texas Health Science Center, Fort Worth, Texas, United States of America; University College London, UNITED KINGDOM

Relationship testing was the first application of DNA-based technologies used for forensic purposes [1]. Indeed, DNA testing has been the gold standard of the forensics applications and greatly impacts society and individuals alike. According to American Association of Blood Banks (AABB) annual reports [2], about 400,000 relationship testing cases are performed each year within the AABB accredited laboratories residing mostly in the United States. This number is likely an underestimate; some laboratories did not report their numbers to AABB, and some laboratories provide services without accreditation. China has a similar number of relationship testing cases as that of the US, and the number of cases has been increasing due to recent national policy change [3]. DNA testings also are applied globally to address issues of fraud and abuse that arise with international migration and refugee family reunions [4]. Recently, US Immigration and Customs Enforcement established DNA testing sites at the southwest border to identify fraudulent familial relationships claimed by immigrants, which may test tens of thousands of cases per year [5]. In addition, many criminal cases and missing person identification cases rely on relationship testing. As of today, it is reasonable to estimate that more than 15 million relationship cases have been tested globally.

With so many cases, it is not uncommon to observe some cases that may have either included unrelated individuals as a certain relationship (i.e., false positive) or excluded truly related individuals as unrelated (i.e., false negative) [6]. A kinship relationship is usually evaluated by comparing the likelihoods of observing the genetic data given 2 alternative hypotheses (i.e., likelihood ratio [LR]): an individual is related to another individual in a defined relationship versus the 2 individuals not related. The higher the LR, the more supported is the proposed relationship. Additionally, the lower the LR (typically <1), the more support there is for the unrelated hypothesis. To render a conclusion about the purported relationship, some laboratories assign a minimum threshold to the LR to support an “inclusion” or “related.” The threshold can vary among laboratories. According to the AABB relationship testing annual report in 2013 [2], 65% of AABB accredited laboratories used an LR threshold of 100 for standard trio cases (i.e., biological mother, child, and alleged father), and 75% of the laboratories used the same LR threshold for parent-child cases (i.e., duo case). Most laboratories report only the LR obtained without a threshold for full-sibling, half-sibling, and other reconstruction cases, although about 20% of the laboratories use an LR threshold of 100 for such cases. Other thresholds (e.g., 10 and 1,000) may be used depending on the proposed relationships [2].

Different laboratories also may implement different conclusion policies for “inclusion,” “exclusion,” and “inconclusive.” Unfortunately, there are no readily available surveys or widely accepted guidelines as to what is deemed “inconclusive” and whether decisions are impacted differently as they approach thresholds, especially for cases with a relatively high LR in an inconclusive range (e.g., 0.01 < LR < 100) but lower than the upper threshold. Although some accredited laboratories may have an internal definition for “inconclusive,” not all users may enact an “inconclusive” range. Moreover, there is no universally accepted inconclusive range. This concern is particularly exacerbated with cases (e.g., border crossing) in which nonscientists who render interpretations may judge an LR that is relatively high but less than the minimum inclusion as non-supportive of parentage and thus as exculpatory or incriminating. The forensic community also recommended [7] that when the LR reaches a certain threshold regardless of the case scenario, an exclusion can be reported, and this policy would apply to criminal paternities. Based on our experience of working with law enforcement and the legal community, the community does consider that, if the LR is not exceedingly large, then the result is weak or exculpatory. Given their assessments, those of the forensic science community, and some laboratory practices, the potential error of using a binary approach of inclusion or exclusion with a single threshold was studied herein. We recognize that not all would use such a binary approach, but the findings still can be illustrative of the consequences for those decision and policy makers that may use a binary or arbitrary interpretation strategy.

There are a number of factors that impact LR calculations, such as the testing hypotheses, the testing kits or markers, the allele frequencies of the relevant population(s), co-ancestry coefficient, the way to address mutations, etc. The selection of commercial kits, and hence the markers and number of markers, is an important factor, as it determines the discrimination power of the test. The kits used in relationship testing could be categorized into 3 generations. Around the early 2000s, most relationship testing laboratories used in-house developed or commercially available kits (e.g., AmpFlSTR Profiler and COfiler) with 8–11 autosomal Short Tandem Repeat (STR) markers per kit. To meet the requisite 13 Combined DNA Index System (CODIS) core loci [8] and to increase discrimination power, multiple kits were used in a testing case. After the early 2000s, kits (e.g., Identifiler, PowerPlex 16) were developed to include all 13 FBI CODIS core loci with a couple of extra markers in a single test. Recently, after the FBI’s mandate to expand the CODIS core loci [9], commercial kits were developed for testing at least 21 autosomal markers (e.g., Globalfiler, PowerPlex Fusion). The first 2 generations of kits (i.e., markers) provided comparable discrimination power, whereas the third-generation (and some second-generation) kits represent current increased capabilities.

Studies [10–11] have evaluated the false conclusion rates in parent-child and full-sibling testings using the 13 CODIS loci. Following the same approach, we evaluated the false conclusion rates of the common relationship testings with the current commercial kits using the common AABB LR thresholds. Pedigrees were simulated as described by Ge and colleagues [10] for various relationships (standard trio, parent-child, full-sibling, half-sibling, first cousin, and unrelated), and LRs were calculated for the simulated relationships using the same method as Ge and colleagues [12]. The details of the methods and results are explained in S1 Text.

The results show that the closer the family relationship is, the higher the accuracy of the test (S1 and S2 Tables). With the Identifiler kit (containing 15 autosomal STR markers), for a trio relationship, using a threshold of a LR of 100 yields a false negative rate (i.e., related identified as unrelated) of 0.058% and a false positive rate (i.e., unrelated identified as related) of 0.0007%. In other words, in 1 in every 1,700 trio cases, a biological father could be falsely identified as unrelated; it is far more unlikely to identify a non-biological father as the biological father (1 in 142,000). Using a different threshold will reduce one false rate but increase the other, with similar markers and methods. For a trio, increasing the LR threshold to 1,000 could reduce the false positive rate to negligible (1 in 500,000) but raises the false negative rate to 0.284% (1 in 350). The false negative and false positive rates for parent-child are higher with a threshold of 100, 1.14% (approximately 1 in 88) and 0.015% (approximately 1 in 6,600), respectively, due to only one reference parent. The relationship testing regarding siblings and cousins had low accuracy with any of these thresholds. Apparently, reporting the LR obtained for distant relationships is based on limited power afforded with current forensic marker panels. A posterior probability of the relationship is calculated (e.g., 99.999%) mostly in civil cases. This value has nothing to do with the accuracy of the test; it is calculated based on the LR and a prior probability, which may be difficult to estimate and can vary by the decision makers, although 50% is used in most civil paternity cases [13].

To estimate the number of cases that could have been falsely excluded or included, assume there have been 10 million relationship cases tested globally with the second-generation kits, a conservative estimate according to [2–3]. The percentage of cases determined as exclusions (i.e., unrelated) has been relatively consistent (approximately 24% to 29% over the years) [2]; it is reasonable to assume an average exclusion rate of 28% (namely, that 72% of the relationship testing cases were truly related), which should be close to the true average exclusion rate, since the error rates for the most commonly test cases are relatively low (Table 1; S2 Table). Based on relationship testing (approximately 40,000 cases) at the Center of Human Identification at the University of North Texas Health Science Center, excluding its specialized missing persons program, about 75% of the cases were trios, 20% were duos, and 5% were other relationships (assumed to be predominantly full-sibling for simplicity and presentation herein). According to the AABB reports [2] and to simplify discussion, 65% of the laboratories use a threshold LR = 100, and 35% use a LR = 1,000 for trio cases; 75% use an LR = 100, and 25% use an LR = 1,000 for parent-child cases; and 20% use LR = 100 for full-sibling cases. With the false rates of given thresholds in S2 Table, the number of falsely concluded cases can be inferred (Table 1). In total, more than 60,000 cases could have been wrongly concluded using the markers in the Identifiler kit. The majority of the false interpretations are related individuals being misinterpreted as unrelated, particularly for the parent-child and more distant relationships. In contrast, it is very unlikely to identify unrelated individuals as close relatives.

**Table 1:** A simplified but reasonable model to estimate the number of falsely interpreted cases with the 15 autosomal markers in the Identifiler kit.

True relationships	No. of cases for each relationship	Proportions of labs adopting a specified LR threshold	False interpretation rates	No. of false interpretations
**Related/inclusion (72%)**	7,500,000 (Trio)	65% (LR = 100)	0.058%	2,036
		35% (LR = 1,000)	0.284%	5,368
	2,000,000 (Parent-child)	75% (LR = 100)	1.14%	12,312
		25% (LR = 1,000)	7.67%	27,612
	500,000 (Full-sibling)	20% (LR = 100)	20.82%	14,990
**Unrelated/exclusion (28%)**	7,500,000 (Trio)	65% (LR = 100)	0.0007%	10
		35% (LR = 1,000)	0.0002%	1
	2,000,000 (Parent-child)	75% (LR = 100)	0.0150%	63
		25% (LR = 1,000)	0.0055%	8
	500,000 (Full-sibling)	20% (LR = 100)	0.032%	9

The false interpretation rates are false negative rates for truly related but excluded as unrelated and false positive rates for unrelated but included as related, respectively.

**Abbreviation**: LR, likelihood ratio

With the third-generation kits (e.g., Globalfiler with 21 STRs), the false rates are significantly reduced compared with the Identifiler kit due to the additional 6 markers. The trio cases, with a threshold of 100, yield false negative and positive rates of 1 in 111,000 and 1 in 10 million, respectively. While reduced rates are achieved with 21 markers, the parent-child and full-sibling cases still have relatively high false negative rates of 1 in 770 and 1 in 160, respectively. Assuming that 5 million cases have been tested with Globalfiler or similar kits globally, there are several thousand cases that could have been wrongly interpreted, predominately for the parent-child or more distant relationships (S3 Table), assuming a binary threshold approach. Therefore, tens or even hundreds of migrant-related relationship cases may be falsely determined as exclusions at the US border DNA testing sites, where tens of thousands of cases are tested in a year (if parent-child is the major case scenario being assessed).

Since not all laboratories or jurisdictions would invoke a binary approach, an arbitrarily inconclusive range of 0.01 < LR < 100 was tested. With this specific inconclusive range, the false negatives would be substantially reduced (i.e., approximately 1 in a million for trios, approximately 2 in 100,000 for parent-child, and 1 in 770 for full-sibling). The false positives remain the same because the inconclusive range is defined below the threshold leading to false positives. Defining and using a reasonable inconclusive category would reduce interpretation errors. However, large proportions of the distant relationships (approximately 71% of half-siblings and 99% of first-cousins) would fall into this inconclusive range. Thus, more distant relationships would be uninformative if an inconclusive range was invoked. Yet still a factor is that nonscientist decision makers are not likely to follow this interpretation approach strictly. Overall, the current STR marker systems—together with the use of a binary decision system with or without an inconclusive category—do not address the overall problem, nor are they more genetically informed.

While these tests evaluate particular hypothesized relationships versus unrelated, the situation is more complicated. People may misstate half-siblings as siblings, or family members may unknowingly assert a sibling relationship that is a half-sibling. In certain countries, cousins could be called siblings (i.e., cultural and social differences). In border control or migration cases, an uncle may claim he is the father of his nephew. Also, the assumption that the parents in trio tests are unrelated may not be true. Genotyping error (e.g., allele dropout or missing data) due to instrument performance or testing kit design is another issue, which may substantially change the LR generated. In such complex scenarios, the chance for a false interpretation can be higher. To overcome these deficiencies, adding more family references and/or more markers (not only autosomal markers, but also X and Y chromosome markers) will reduce the false interpretation rate.

The most recent development of relationship testing, particularly by the direct-to-consumer testing companies, is to use high-density Single Nucleotide Polymorphism (SNP) panels from chip arrays (e.g., approximately 800 K SNPs) or from whole-genome sequencing data to measure the genomic regions with identity-by-descent (IBD) and determine even very distant relationships [14]. Theoretically, with so much SNP data, the accuracy of close relationship testings should approach 100% (which could rectify a serious concern of misidentifications both related and unrelated), and third-degree or more distant relationships may be determined with high accuracy [14]. This increase in genetic data is the engine of forensic genetic genealogy which recently has helped solve some well-noted cases [15]. Moreover, the much greater amount of data can better address the uncertainties that may occur with wrongly stated or alleged relationships and cultural differences that may contribute to incorrectly stated kinships. While we embrace and advocate dense SNP analyses, this technology has not been systematically validated for forensic or relationship testing purposes, such as the accuracies for different relationships, the variance, technology limitations, etc. Validation studies with forensic relevance should be sought with all due speed as this technology is the future of relationship testing to substantially reduce false interpretations. With solid validation, high-density SNP panel-based technologies could meet scientific scrutiny as well as the Frye or Daubert (legal) admissibility standards, in a similar fashion to that of current forensic DNA testing systems. We encourage the relationship testing community to move from traditional STR-based technology to high-density SNP-based technology with the following actionable recommendations.

1. Address the issues related to current STR-based technology by
  - Better defining the inconclusive category and what it should mean
  - Better educating and training decision makers on the interpretation of the testing results and the limitation of the STR technology
2. Start to implement high-density SNP-based technology to reduce inconclusive and error rates obtained from traditional relationship testing cases by
  - Systematically validating the high-density SNP-based methods for common and distant relationship testing in terms of accuracies, variance, limitations, and interpretation guidelines
  - Developing laboratory-friendly standard workflow and software tools for high-density SNP-based relationship testing and making them readily accessible to the community
  - Educating the user communities on the strengths and limitations of high-density SNP-based methods, as well as the interpretation of the testing results

## Supporting information

S1 Text The methods and results of the simulation study. (DOCX)

S1 Table The counts of LR per range of LRs for (a) the true standard trio (“Trio”), parent-child (“PC”), full-sibling (“FS”), half-sibling (“HS”), and first cousin (“CS”) relationships calculated as the same relationships and (b) unrelated calculated as related, with Identifiler and Globalfiler kits. Each relationship (Trio, PC, FS, HS, CS, and unrelated) was simulated 10 million times. In (a), the true relationships (e.g., trio) were simulated, and the LRs of the simulated relationships were calculated based on two hypotheses, the same true relationship (e.g., trio) versus unrelated. In (b), the unrelated relationships were simulated, and the LRs of the simulated relationship were calculated based on two hypotheses, an alleged relationship (e.g., trio) versus unrelated.
(DOCX)

S2 Table The false rates of the LR per range of LRs for (a) the true standard trio (“Trio”), parent-child (“PC”), full-sibling (“FS”), half-sibling (“HS”), and first cousin (“CS”) relationships calculated as the same relationships and (b) unrelated calculated as related, with Identifiler and Globalfiler kits. In (a), the true relationships (e.g., trio) were simulated, and the LRs of the simulated relationship were calculated based on two hypotheses, the same true relationship (e.g., trio) versus unrelated. In (b), the unrelated relationships were simulated, and the LRs of the simulated relationship were calculated based on two hypotheses, an alleged relationship (e.g., trio) versus unrelated.
(DOCX)

S3 Table A simplified but reasonable binary model to estimate the number of falsely interpreted cases with the 21 autosomal markers using the Globalfiler kit. The false interpretation rates are false negative rates for truly related but excluded as unrelated and false positive rates for unrelated but included as related, respectively.
(DOCX)
