# Supplementary material for: How many familial relationship testing results could be wrong?
Source: PLoS Genet. 2020 Aug 13;16(8):e1008929. doi: 10.1371/journal.pgen.1008929 (PMC7425842; doi:10.1371/journal.pgen.1008929)
Supplement: S1 Text — (DOCX) [file pgen.1008929.s001.docx]

**S1 Text. The methods and results of the simulation study**

**Methods**

A simulation study was performed to estimate the false positive and false negative rates of common familial relationship testing cases based on a binary inclusion/exclusion threshold. 10 million standard trio, parent-child, full-sibling, half-sibling, first-cousin, and unrelated DNA profiles were simulated using Caucasian population data [S1] with the autosomal markers contained within the commercially available STR Kits (i.e., Identifiler and Globalfiler). These six relationships were selected as they are the most common relationship testing cases. The software program MPKin was used to simulate the pedigrees and DNA profiles [S2]. In the simulation, the genotypes of founders (i.e., individuals without parents in the pedigree) were randomly assigned according to the conditional genotype frequencies given observed alleles of the founders of each locus, and each locus was treated independently. The conditional genotype frequencies were calculated by the procedure described in Balding and Nichols [S3] with a co-ancestry coefficient θ = 0.01 as recommended by the NRCII Report [S4]. Founders transmitted with equal probability a single allele at each locus to his/her child. Mutations during the transmissions were allowed according to the two-phase model [S2], which is similar to the step-wise mutation model used by many laboratories. Both paternal and maternal mutation rates were used according to the AABB [S5]. This study used allele frequency data from the Caucasian population for illustrative purposes.

In this study, the autosomal short tandem repeat (STR) markers of the Identifiler [S6] and Globalfiler [S7] kits were simulated as representatives of second and third generations of forensic STR kits. Applied Biosystem’s Identifiler and Promega’s PowerPlex 16 represent the major second generation forensic STR kits. Since their launch in the early 2000s, these kits have been widely used for forensic and paternity testing cases. The Identifiler and PowerPlex 16 kits share 13 of their 15 STR markers [S8], and the powers of discrimination of these two kits are comparable. Applied Biosystem Globalfiler and Promega PowerPlex Fusion were launched in 2012 as the third generation of commercial STR kits to meet the requirement of the FBI CODIS core loci expansion [S9]. The marker selections of these two kits overlap, and the powers of discrimination are also comparable.

The likelihood ratios (LRs) given defined hypotheses (e.g., two profiles are parent-child relationship vs. they are unrelated) were calculated using the methods described in [S2]. All autosomal markers were treated as independent, θ = 0.01 was used in all LR calculations, and the two-phase mutation model is incorporated in calculations.

**Results**

S1 Table displays the counts of LRs in each LR range for the common relationships, either the true relationship identified as the same true relationship or as unrelated, with each second and third generation kit (Identifiler or Globalfiler kits, respectively). S2 Table shows the same data as in S1 Table but instead in the format of percentages.

As expected, in general the closer the relationships are, the higher are the LRs. The majority of the LRs of close relationships (i.e., trio, parent-child, and full-siblings) are higher than a LR of 100 (the most commonly used threshold), and only a small percentage of LRs of the distant relationships (i.e., half-siblings and first-cousin) are higher than a LR of 100, for both Identifiler or Globalfiler kits (Table S1.a). These results indicate that the current commercial STR kits could identify close relationships with a relatively low error rate, but not so with more distant relationships. The LRs with the Globalfiler kit are typically higher than those obtained with the Identifiler kit, because of six additional markers. Particularly for trios, the Globalfiler kit approaches 100% accuracy for the trio relationship.

With a binary LR threshold of 100, the chances that unrelated individuals are identified as related are much smaller than the chances of identifying true related (S1.b Table). The concern though is for the false negatives (i.e., related identified as unrelated) than for the false positives (i.e., unrelated identified as related). There have been some reports on false positives [S10-S11], but the reports of false negatives tend to focus on non-scientific error, such as a possible father submitting someone else’s DNA for testing. The false negative rate of DNA testing for kinship analysis has not been adequately addressed.

S3 Table shows the estimation of the numbers of false interpreted cases with 21 autosomal markers in the Globalfiler kit, following the same model as for Table 1. Since the third generation kits (Globalfiler or similar kits) were launched in 2012, approximately 5 million relationship cases were tested globally (a reasonable estimation). Assuming the same proportions of each relationship in tests and the same proportions of labs adopting a specified LR threshold as in Table 1, there are several thousand true relationship cases that could have been wrongly interpreted as unrelated, predominately for the parent-child or more distant relationships using a binary approach. False positive interpretations with the Globalfiler kit are rare.

**References**

S1. Moretti TR, Moreno LI, Smerick JB, Pignone ML, Hizon R, Buckleton JS, et al. Population data on the expanded CODIS core STR loci for eleven populations of significance for forensic DNA analyses in the United States. Forensic Science International: Genetics. 2016;25(Supplement C):175-81.

S2. Ge J, Budowle B, Chakraborty R. DNA identification by pedigree likelihood ratio accommodating population substructure and mutations. Investig Genet. 2010;1(1):8.

S3. Balding DJ, Nichols RA. DNA profile match probability calculation: how to allow for population stratification, relatedness, database selection and single bands. Forensic Science International. 1994;64(2-3):125-40.

S4. National Research Council Committee on DNA Forensic Science: An Update. The Evaluation of Forensic DNA Evidence (1996) National Academy Press, Washington DC

S5. AABB Relationship Testing Annual Reports 2008, http://www.aabb.org/sa/facilities/Documents/rtannrpt08.pdf. Accessed May 23, 2020.

S6. Applied BioSystems, AmpFlSTR Identifiler PCR Amplification Kit User guide (2012)

S7. Applied BioSystems, AmpFlSTR Globalfiler PCR Amplification Kit User guide (2016).

S8. STRBase, https://strbase.nist.gov/multiplx.htm. Accessed May 23, 2020.

S9. Hares DR. Expanding the CODIS core loci in the United States. Forensic Science International: Genetics. 2012;6(1):e52-e4.

S10. Li L, Ge J, Zhang S, Guo J, Zhao S, Li C, et al. Maternity exclusion with a very high autosomal STRs kinship index. International journal of legal medicine. 2012;126(4):645-8.

S11. The Associated Press, “Lawsuit: Paternity test falsely ID'd Baltimore man as father” (16 December 2019). https://abcnews.go.com/Weird/wireStory/lawsuit-paternity-test-falsely-idd-baltimore-man-father-67751109. Accessed May 23, 2020.
