# Supplementary material for: How many familial relationship testing results could be wrong?
Source: PLoS Genet. 2020 Aug 13;16(8):e1008929. doi: 10.1371/journal.pgen.1008929 (PMC7425842; doi:10.1371/journal.pgen.1008929)
Supplement: S1 Table — Each relationship (Trio, PC, FS, HS, CS, and unrelated) was simulated 10 million times. In (a), the true relationships (e.g., trio) were simulated, and the LRs of the simulated relationships were calculated based on two hypotheses, the same true relationship (e.g., trio) versus unrelated. In (b), the unrelated relationships were simulated, and the LRs of the simulated relationship were calculated based on two hypotheses, an alleged relationship (e.g., trio) versus unrelated. (DOCX) [file pgen.1008929.s002.docx]

S1 Table. The counts of LR per range of LRs for (a) the true standard trio (Trio), parent-child (PC), full-sibling (FS), half-sibling (HS), and first-cousin (CS) relationships calculated as the same relationships and (b) unrelated calculated as related, with Identifiler and Globalfiler kits. Each relationship (Trio, PC, FS, HS, CS, and unrelated) was simulated 10 million times. In (a), the true relationships (e.g., trio) were simulated and the LRs of the simulated relationships were calculated based on two hypotheses, the same true relationship (e.g., trio) vs. unrelated. In (b), the unrelated relationships were simulated and the LRs of the simulated relationship were calculated based on two hypotheses, an alleged relationship (e.g., trio) vs. unrelated.

1. The counts of LR for the scenarios of true relationships calculated as the same relationships

| LR | Identifiler | | | | | Globalfiler | | | | |
| --- | --- | --- | --- | --- | --- | --- | --- | --- | --- | --- |
|  | Trio | PC | FS | HS | CS | Trio | PC | FS | HS | CS |
| 0<LR<0.01 | 13 | 158 | 12,951 | 8,331 | 184,519 | 0 | 6 | 6,297 | 18,084 | 34 |
| 0.01<LR<1 | 276 | 6,237 | 281,548 | 1,789,251 | 5,330,082 | 3 | 269 | 92,834 | 1,239,280 | 3,245,180 |
| 1≤LR<10 | 764 | 26,750 | 597,934 | 3,849,794 | 3,307,324 | 12 | 1,124 | 192,188 | 2,599,105 | 5,475,193 |
| 10≤LR<100 | 4,725 | 81,346 | 1,189,757 | 3,262,506 | 1,047,021 | 43 | 6,545 | 425,764 | 3,264,803 | 1,229,494 |
| 100≤LR<1000 | 22,620 | 652,170 | 1,780,748 | 977,346 | 125,009 | 259 | 29,716 | 778,115 | 2,083,485 | 49,635 |
| 1000≤LR | 9,971,602 | 9,233,339 | 6,137,062 | 112,772 | 6045 | 9,999,683 | 9,962,340 | 8,504,802 | 795,243 | 464 |

1. The counts of LR for the scenarios of truly unrelated calculated as related (Trio, PC, FS, HS, or CS)

| LR | Identifiler | | | | | Globalfiler | | | | |
| --- | --- | --- | --- | --- | --- | --- | --- | --- | --- | --- |
|  | Trio | PC | FS | HS | CS | Trio | PC | FS | HS | CS |
| 0<LR<0.01 | 9,998,754 | 9,972,234 | 8,333,919 | 1,433,116 | 1,404,745 | 9,999,990 | 9,998,786 | 9,581,103 | 3,920,149 | 3,422 |
| 0.01<LR<1 | 976 | 20,137 | 1,533,610 | 7,533,728 | 7,538,083 | 6 | 946 | 388,944 | 5,527,229 | 8,315,437 |
| 1≤LR<10 | 127 | 4782 | 108,623 | 947,655 | 968,412 | 1 | 138 | 23,935 | 489,808 | 1,645,080 |
| 10≤LR<100 | 71 | 1304 | 20,610 | 83,223 | 86316 | 2 | 82 | 5,012 | 59,268 | 35,965 |
| 100≤LR<1000 | 48 | 996 | 2,861 | 2261 | 2424 | 0 | 32 | 859 | 3446 | 95 |
| 1000≤LR | 24 | 547 | 377 | 17 | 20 | 1 | 16 | 147 | 100 | 1 |
