# Supplementary material for: How many familial relationship testing results could be wrong?
Source: PLoS Genet. 2020 Aug 13;16(8):e1008929. doi: 10.1371/journal.pgen.1008929 (PMC7425842; doi:10.1371/journal.pgen.1008929)
Supplement: S2 Table — In (a), the true relationships (e.g., trio) were simulated, and the LRs of the simulated relationship were calculated based on two hypotheses, the same true relationship (e.g., trio) versus unrelated. In (b), the unrelated relationships were simulated, and the LRs of the simulated relationship were calculated based on two hypotheses, an alleged relationship (e.g., trio) versus unrelated. (DOCX) [file pgen.1008929.s003.docx]

S2 Table. The false rates of the LR per range of LRs for (a) the true standard trio (Trio), parent-child (PC), full-sibling (FS), half-sibling (HS), and first-cousin (CS) relationships calculated as the same relationships and (b) unrelated calculated as related, with Identifiler and Globalfiler. In (a), the true relationships (e.g., trio) were simulated and the LRs of the simulated relationship were calculated based on two hypotheses, the same true relationship (e.g., trio) vs. unrelated. In (b), the unrelated relationships were simulated and the LRs of the simulated relationship were calculated based on two hypotheses, an alleged relationship (e.g., trio) vs. unrelated.

1. The false negative rates for the scenarios of true relationships calculated as the same true relationships

| LR | Identifiler | | | | | Globalfiler | | | | |
| --- | --- | --- | --- | --- | --- | --- | --- | --- | --- | --- |
|  | Trio | PC | FS | HS | CS | Trio | PC | FS | HS | CS |
| <10 | 0.011% | 0.33% | 8.92% | 56.47% | 88.22% | 0.0002% | 0.01% | 2.91% | 38.56% | 87.20% |
| <100 | 0.058% | 1.14% | 20.82% | 89.10% | 98.69% | 0.0006% | 0.08% | 7.17% | 71.21% | 99.50% |
| <1000 | 0.284% | 7.67% | 38.63% | 98.87% | 99.94% | 0.0032% | 0.38% | 14.95% | 92.05% | 100.00% |

1. The false positive rates for the scenarios of truly unrelated calculated as related (Trio, PC, FS, HS, or CS)

| LR | Identifiler | | | | | Globalfiler | | | | |
| --- | --- | --- | --- | --- | --- | --- | --- | --- | --- | --- |
|  | Trio | PC | FS | HS | CS | Trio | PC | FS | HS | CS |
| >1000 | 0.0002% | 0.0055% | 0.0038% | 0.0002% | 0.0002% | 0.00001% | 0.0002% | 0.0015% | 0.001% | 0.00001% |
| >100 | 0.0007% | 0.015% | 0.032% | 0.023% | 0.024% | 0.00001% | 0.0005% | 0.010% | 0.035% | 0.001% |
| >10 | 0.0014% | 0.029% | 0.24% | 0.86% | 0.89% | 0.00003% | 0.0013% | 0.06% | 0.63% | 0.36% |
