# Supplementary material for: How many familial relationship testing results could be wrong?
Source: PLoS Genet. 2020 Aug 13;16(8):e1008929. doi: 10.1371/journal.pgen.1008929 (PMC7425842; doi:10.1371/journal.pgen.1008929)
Supplement: S3 Table — The false interpretation rates are false negative rates for truly related but excluded as unrelated and false positive rates for unrelated but included as related, respectively. (DOCX) [file pgen.1008929.s004.docx]

S3 Table. A simplified but reasonable binary model to estimate the number of falsely interpreted cases with the 21 autosomal markers in the Globalfiler kit. The false interpretation rates are false negatives rates for truly related exclude as unrelated and false positive rates for unrelated included as related, respectively.

| True relationships | No. of cases for each relationship | Proportions of labs adopting a specified LR threshold | False interpretation rates | No. of false interpretations |
| --- | --- | --- | --- | --- |
| Related  /Inclusion  (72%) | 3,7500,000  (Trio) | 65% (LR=100) | 0.0006% | 11 |
|  |  | 35% (LR=1,000) | 0.0032% | 30 |
|  | 1,000,000 (Parent-Child) | 75% (LR=100) | 0.08% | 432 |
|  |  | 25% (LR=1,000) | 0.38% | 684 |
|  | 250,000  (Full-sibling) | 20% (LR=100) | 7.17% | 2,581 |
| Unrelated /Exclusion  (28%) | 3,7500,000  (Trio) | 65% (LR=100) | 0.00001% | 0 |
|  |  | 35% (LR=1,000) | 0.00001% | 0 |
|  | 1,000,000 (Parent-Child) | 75% (LR=100) | 0.0005% | 1 |
|  |  | 25% (LR=1,000) | 0.0002% | 0 |
|  | 250,000  (Full-sibling) | 20% (LR=100) | 0.010% | 1 |
